# Circulating Cell-Free DNA in Inflammatory Bowel Disease: Liquid Biopsies with Mechanistic and Translational Implications

**DOI:** 10.12703/r/12-14

**Published:** 2023-06-14

**Authors:** Cher Shiong Chuah, Lena Fischer, Gwo-Tzer Ho

**Affiliations:** 1 Edinburgh IBD Science Unit, Centre for Inflammation Research, University of Edinburgh

**Keywords:** IBD, UC, CD, cfDNA, mitochondria, liquid biopsy

## Abstract

This review examines the role of circulating cell-free DNA (cfDNA) as potential drivers of inflammation and their potential application as mechanistic biomarkers in Inflammatory Bowel Diseases (IBD). These DNA fragments contain significant information about their origins, the underlying host pathology leading to their release, and possess properties that can fuel the inflammatory process. Recent advances in sequencing and analytical approaches have made the translation of cfDNA into clinical practice a promising prospect. We focus on the functional relevance of cfDNA in the inflammatory process and discuss its potential for future assessments of IBD activity and identification of therapeutic options.

## Introduction

Inflammatory bowel diseases (IBD), Ulcerative colitis (UC) and Crohn’s disease (CD) are chronic immune-mediated conditions with complex pathogenic mechanisms that drive the abnormal process of gut mucosal inflammation. Genetic susceptibility (>300 genetic loci), environmental influences (such as diet, drugs, and smoking), altered microbiome, and dysregulated immune and gut barrier functions are key contributory factors to the development of IBD. With recent scientific advances, the quest for precision medicine to predict outcomes and select the right treatment has provided much promise but realistic clinical translation opportunities have not been forthcoming^1^. Rapid progress in identifying new potential drug targets has shifted the challenge of drug discovery towards identifying dominant inflammatory mechanisms within the heterogeneous nature of IBD to facilitate decision-making in selecting the right drug for the right patient. In this context, increasingly deeper multi-omic data in very large patient groups combined with powerful computational approaches will help, but the question remains as to whether there are entirely underexplored areas in inflammation biology that have not been fully appreciated.

Our review focuses on the roles of circulating cell-free DNA (cfDNA) as potential drivers and mechanistic biomarkers and their development as ‘liquid biopsies’ in IBD. We recently showed that circulating mitochondrial DNA could be found in active IBD^2^, an interesting observation in line with other conditions where liberated host DNA triggers inflammation, in a spectrum of conditions ranging from systemic inflammatory syndrome (SIRS) to chronic immune-mediated diseases, such as systemic lupus erythematosus (SLE). Furthermore, DNA complexed with proteins can be pathogenic drivers of immune-mediated diseases, such as psoriasis^3,4^. Recently, therapeutic developments of nucleic acid-based therapies using oligonucleotides to gene targets, such as SMAD7, to mimic the effect of bacterial DNA or to influence specific splicing of anti-inflammatory microRNA miR-124 by small-molecule ABX464 have highlighted the translational importance of such approaches in IBD (Table 1)^5,6^. In other disease areas, mRNA vaccines for COVID-19 and the use of circulating tumour DNA in oncology are in clinical use^7^. However, DNA pathways and translational technologies are virtually unexplored areas in IBD. Here, we present a comprehensive appraisal of circulating nucleic acids (with a particular focus on DNA) and their potential roles in IBD with a focus on translational concepts and opportunities.

**Box 1. Key milestones in the development of cfDNA technology**

**1948**      Mandel and Metais described the presence of DNA in blood

**1966**      Elevated circulating DNA in SLE with anti-DNA antibody formation

**1973**      Presence of circulating tumour DNA (ctDNA) in cancer patients – ‘Liquid biopsy’

**1990**      Launch of the human genome project

**1997**      Discovery of circulating cell-free foetal DNA in maternal plasma

**2001**      First draft of human genome published

**2004**      First metagenomics study

**2008**      First successes of prenatal diagnosis using foetal cfDNA in maternal plasma

**2010**      High circulating mitochondrial DNA in non-infective Systemic Inflammatory Response Syndrome (SIRS).

**2018**      High circulating mitochondrial DNA in active IBD, a chronic inflammatory condition.

**2020**      The Galleri blood test to screen for ~50 cancer ctDNA in 165 000 patients in England.

**Table 1.** Nucleic acid-based therapies reaching human trials in IBD.

Name	Disease	Mechanism	Target Development Progress	Reference
Cobitolimod	Ulcerative colitis	DNA-based oligonucleotide activating TLR-9 receptor leading to upregulation of anti-inflammatory IL10+ macrophages and regulatory T cells and suppresses Th17 cells	Phase 2b completed in 2020. Phase 3 trials underway	5,6
Mongersen	Crohn’s disease	Oral Smad7 antisense oligonucleotide	Phase 3 completed in 2020. No efficacy vs placebo in active Crohn’s disease.	8
ABX-464	Ulcerative colitis	Selectively upregulates miR-124 in immune cells	Phase 2b completed. Phase 3 trials are in planning phase.	9,10
Alicaforsen	Crohn’s disease Ulcerative colitis	Antisense oligonucleotide inhibiting ICAM-1	Phase 3 is completed. No efficacy in Crohn’s. Limited efficacy in ulcerative proctitis	11,12

## Discovery of circulating DNA – Origins

DNA, the blueprint of all biological information, is contained in specialised compartments - in the nucleus or intracellular mitochondria, separate from the rest of the eukaryotic cell. This partitions major DNA functions and cellular processes, including gene expression, genome replication, and the repair of damaged DNA. Multiple biological machineries exist to tightly regulate and maintain a finely tuned state of cellular and tissue homeostasis, where cells can replicate while safely transferring their DNA.

cfDNA is present in the circulating plasma, urine, and other bodily fluids in humans^13^. It comprises double-stranded DNA fragments that are overwhelmingly short, <200 base pairs (bp), and are normally present at low concentrations^14^. The circulation of cfDNA in blood was first described in 1948, shortly after World War II, by Mandel and Metais^15^ using a perchloric acid precipitation method^16^. It is interesting to note that this was only a few years after DNA was first demonstrated as the material of inheritance and preceded Watson and Crick’s seminal paper on the double-helical structure of DNA^17^. This discovery received little attention during that era, and it was not until 1966 that it was found that cfDNA levels were elevated in systemic lupus erythematosus, a disease characterised by the formation of anti-dsDNA antibodies^18^. Interest in cfDNA began to increase when it was shown to be elevated in cancer patients in 1973, with cancer patients having approximately 10 times more cfDNA than healthy controls^19^. It has only been in the last 10–20 years that improved technology has led to an explosion of interest in cfDNA, particularly in the field of oncology. More recently, there has been emerging evidence that cfDNA may play an important role in regulating immune responses in health and various disease states (Table 2).

**Table 2.** Summary of clinical studies of cell-free DNA and behaviour in various disease states.

Disease State	cfDNA subtype	Sample Size	Effects	References
Trauma	mtDNA	86 patients 40 controls	mtDNA was associated with risk of post-trauma SIRS	20
Trauma	mtDNA	15 patients 12 controls	mtDNA activates PMNs through TLR9	21
Trauma	mtDNA, ncDNA	104 patients 42 controls	No association between mtDNA and injury severity. ncDNA correlates but does not directly contribute to immune response.	22
Systemic Lupus Erythematosus	Total cfDNA, mtDNA, ncDNA	43 patients 50 controls	Total cfDNA higher in SLE Relative mtDNA higher in SLE. Higher fragmentation in SLE.	23
Systemic Lupus Erythematosus	Total cfDNA	52 patients 25 controls	cfDNA correlates with CRP cfDNA negatively correlates with anti-dsDNA, ANA	24
Systemic Lupus Erythematosus	Total cfDNA	58 patients 259 controls	cfDNA higher in SLE cfDNA higher in active disease	25
Rheumatoid Arthritis	mtDNA, ncDNA	74 patients 63 controls	ncDNA higher in RhA mtDNA no association in RhA	26
Rheumatoid Arthritis	mtDNA, ncDNA	32 patients, longitudinal sampling at 3 and 6 months, 0 controls	ncDNA and mtDNA decreased significantly following treatment	27
Rheumatoid Arthritis	Total cfDNA	30 patients 21 controls	cfDNA higher in RhA.	28
Sepsis	Total cfDNA	27 sepsis 7 trauma	cfDNA higher in sepsis vs trauma	29
Sepsis	Total cfDNA	80 patients 14 controls	cfDNA higher in non-survivors	30
Sepsis	Total cfDNA	255 patients 0 controls	cfDNA higher in non-survivors	31
Sepsis	mtDNA	20 sepsis 40 post-surgery 20 controls	mtDNA higher in sepsis vs post-surgical inflammation	32
COVID-19	mtDNA	97 patients 0 controls	mtDNA higher in non-survivors	33
Major depression	mtDNA	109 major depressive disorder 28 bipolar disorder 17 schizophrenia 29 controls	mtDNA decrease in depression and bipolar disorders	34

*cfDNA – cell-free DNA; mtDNA – mitochondrial cell-free DNA; ncDNA – nuclear cell-free DNA; SIRS – systemic inflammatory response syndrome; PMNs – polymorphonuclear neutrophils; TLR9 – toll-like receptor-9; SLE – systemic lupus erythematosus; CRP – C-reactive protein; RhA – rheumatoid arthritis*

cfDNA in blood plasma consists of a mixture of fragmented DNA molecules released from various tissues within the body. They are primarily nucleosome-associated fragments that are wrapped around histone proteins, such as spools rather than free strands of DNA^35^. Each cfDNA fragment bears the molecular signatures of its cell of origin, such as DNA methylation status. The cellular/tissue origins of cfDNA have been studied using several approaches, such as nucleosome positioning^35,36^ and methylation analysis^37,38^.

cfDNA mostly originates from haematopoietic cell death^37,39–41^. In health, Moss et al. estimated that cfDNA is derived from 55% white blood cells, 30% erythrocyte progenitors, and 10% vascular endothelial cells, accounting for approximately 95% of cfDNA^37^. Of the remainder of cfDNA from non-haematopoietic tissue, the liver is estimated to account for a further 1–1.3% of the total cfDNA, which is consistent with estimates of daily liver cell turnover via apoptosis^38,42^. Notably, the absence of cfDNA signals from other tissues in the body with a high turnover rate, such as intestinal epithelial cells, lung, kidney, and skin, may reflect alternative clearance routes.

In disease states such as sepsis and cirrhosis, the elevation in cfDNA is mostly derived from blood cells^37,38^. Owing to tight nucleosome-wrapping of cfDNA and generally low quantities in circulation, it is unclear if they impart a direct inflammatory impact. However, circulating mitochondrial DNA exists in short fragments that have a strong immunostimulatory effect and from this perspective, mitochondrial DNA has received much recent attention in inflammatory diseases^43^.

## Circulating cfDNA as an inflammatory trigger – ‘Matzinger’s Danger Model’

In 2000, Polly Matzinger proposed the ‘Danger Model’, where our immune system is primarily geared towards recognising and responding to danger irrespective of its origins. Hence, the immune system can be rapidly mobilised to respond to pathogen-associated molecular patterns (PAMPs) when host defences are compromised (e.g. in septicaemia) and danger-associated molecular patterns (DAMPs) are released during significant tissue damage. Although the former is apposite in IBD, given its close juxtaposition with gut microbiota, endogenously released DAMPs may also be important^44^. In both settings, activation of pattern recognition receptors (PRRs) by PAMPs or DAMPs leads to inflammation^45^.

Nucleic acids, whether released from our cells or a foreign source (e.g. bacteria, viruses, and vaccines), encapsulate Matzinger’s dictum, in that our immune system has evolved to develop built-in ‘alarm systems’ to respond to intrusion and calibrate an appropriate response. Following significant tissue damage in major trauma, Hauser et al. showed that mitochondrial DAMPs, such as formylated peptides and mitochondrial DNA, are released into the circulation with a direct functional effect of activating the immune system, leading to systemic inflammatory response syndrome (SIRS)^21^. SIRS is a serious condition characterised by systemic inflammation (fever, tachycardia, and breathlessness), multi-organ dysfunction and failure associated with high morbidity and mortality. In patients with severe trauma, mitochondrial cfDNA was thousands of folds higher than in healthy controls and persisted for 24 hours after injury. The authors further showed that the strong immune response was mediated by the activation of TLR9 by mitochondrial cfDNA in neutrophils.

We have shown that elevated mitochondrial cfDNA can be detected in the blood of patients with active and extensive IBD^2^. In fulminant cases of IBD, patients can present with evidence of systemic inflammation (such as SIRS) without evidence of bacterial infection. In our study, a subset of patients with severe IBD showed significantly decreased mitochondrial cfDNA levels after the removal of the inflamed colon^2^. Overall, circulating mitochondrial cfDNA has been detected in many inflammatory conditions and, most recently, is associated with disease severity in COVID-19^33^.

## Circulating cell-free mitochondrial versus genomic DNA

Mitochondria descended from ɑ-proteobacteria in the ancestry of eukaryotic cells 1.5 billion years ago^46^, and consequently, mitochondrial cfDNA possesses unmethylated CpG motifs similar to bacterial DNA^47^. CpG motifs are short fragments of DNA that have cytosine followed by guanine residues, and they have particular significance in stimulating immune responses, notably (but not exclusively) via the Toll-like Receptor 9 (TLR9) signalling pathway^48^. Mitochondrial cfDNA is shorter (40–60 bp), more fragmented, has a higher GC content, and is not bound to nucleosomes compared to nuclear cfDNA^49^.

On the other hand, genomic DNA generally circulates with a size profile in the region of 20–200 bp with a peak of approximately 166 bp, corresponding to the size of a nucleosome. Other peaks were noted at ~320 bp and ~1000 bp, corresponding to dinucleosomes and chromatin, respectively^50^.

The innate immune system has developed a tiered system of nucleic acid sensors to recognise and respond to mislocalised or foreign DNA^51^, which is strategically located in cell compartments free of resident DNA (e.g. cytoplasm) to detect tissue injury and infection^51^. There are three main DNA sensors: TLR9^52^, cyclic GMP-AMP synthase (cGAS), and Absent in Melanoma 2 (AIM2). TLR9 detects DNA rich in unmethylated CpG dinucleotides, which are rare in mammalian genomes, but are common in microbial and mitochondrial DNA. cGAS binds double-stranded DNA leading to activation of STING (stimulator of interferon genes) and resulting in type I interferon release, and the activation of NF-κB pathway to drive the secretion of inflammatory cytokines, such as tumour necrosis factor (TNF) and interleukin-6 (IL-6)^53^. AIM2 signalling leads to inflammasome activity and the cleaving of pro–IL-1β and pro–IL-18 to enable the secretion of the active forms of these inflammatory cytokines and also drives an inflammatory program of cell lysis (called pyroptosis^54^). A diverse range of noxious stimuli by damaging the mitochondria can result in mitochondrial DNA release that triggers NLRP3 inflammasome activation, thus acting as a nexus for the cellular ‘alarm system’^55^. TLR9, cGAS and AIM2 have different nucleic acid-sensing capacities based on CpG methylation, strand, and fragment length; however, it is notable that these receptors are located within the cellular cytosol (or endosomes in the case of TLR9) and are expressed mainly in immune cells. Hence, the entry mechanisms and types of immune cells involved are important for circulating and extracellular DNA.

While naked cfDNA is easily degraded, oxidation induces structural changes, which make the DNA more resistant to degradation and, therefore, available for uptake^56^. This is particularly relevant for mitochondrial DNA, where high reactive oxygen species generation leads to DNA oxidation and thereby significantly confers its inflammatory potential to NLRP3-inflammasome activation. Other entry mechanisms exist; for example, a process mediated by the antimicrobial peptide LL37 that can bind to both genomic and mitochondrial DNA. When bound to LL37, cfDNA condenses and forms a stable complex that is resistant to nuclease degradation^57^. DNA-LL37 complexes can then be transported via lipid rafts into the endosomal component of plasmacytoid dendritic cells, for example, where they can activate TLR9 and subsequently induce a type 1 interferon response^58^. This process can be pathogenic and is implicated in a range of diseases, including type 1 diabetes^59^, atherosclerosis^57,60^, psoriasis^4^, and SLE^61^.

## Circulating cfDNA release and clearance

**Figure 1.**
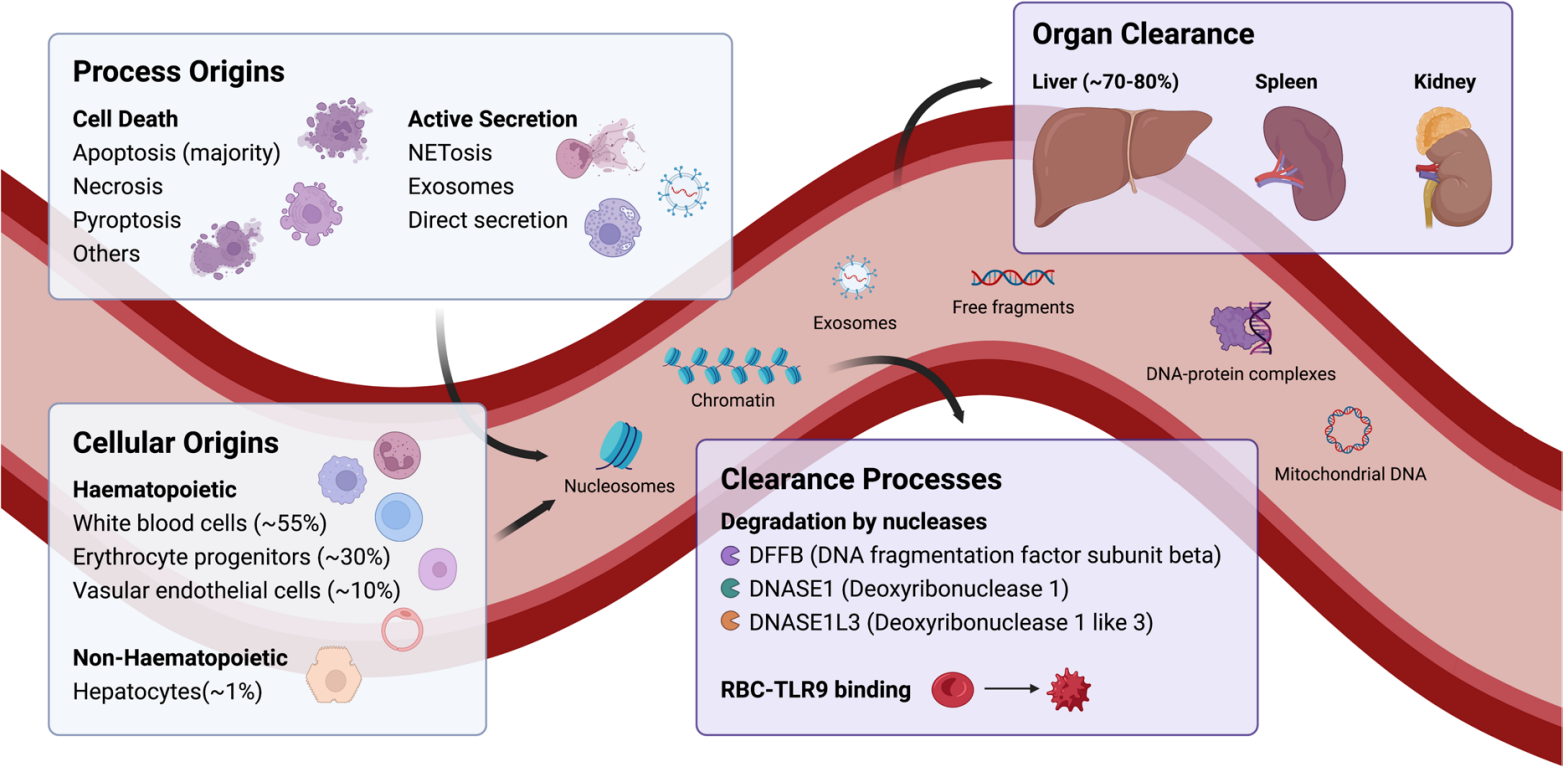
Lifecycle of circulating cell-free DNA.

Figure 1 summarises the current understanding of the life cycle of cfDNA. cfDNA is released during cell death and/or active secretion. Cell death processes include apoptosis, necrosis, mitotic catastrophe, autophagy, necroptosis and pyroptosis, whereas active secretory processes include active secretion of cfDNA, packaging and circulation within exosomes and release in processes such as NETosis^62^. In addition, cfDNA can arise from exogenous sources, such as bacterial and viral DNA, during infection. In healthy states, the majority of cfDNA is packaged into nucleosomes and released as a by-product during apoptosis. Conversely, in disease states, uncontrolled cell death from necrosis can occur leading to the release of long fragments of 10,000 bp^63,64^. Therefore, the balance between apoptosis and necrosis can alter the cfDNA fragment size profile and indicate the underlying pathological processes. In severe trauma, nuclear cfDNA, but not mitochondrial cfDNA, correlates with early host inflammatory response^22^. Conversely, there is a higher mitochondrial/nuclear cfDNA ratio in patients with sickle cell disease than in healthy controls^65^.

The half-life of cfDNA is short and is generally estimated to range from a few minutes to 1–2 hours^66–69^. Different clearance processes are likely to account for this variability. For example, studies examining the clearance of foetal DNA from maternal plasma show a biphasic process consisting of a rapid phase with a half-life of 10–60 mins and a slow phase with a half-life of 13 hours^70^. In cancer, cfDNA has an estimated half-life of 1–2 hours^71,72^. Several factors are known to affect cfDNA clearance, including the formation of molecular complexes which reduce degradation, epigenetic regulation, levels of nucleases, and transport mechanisms within the liver, spleen, and kidneys that remove cfDNA. These factors are not fully understood. However, emerging evidence suggests that these processes are not random^73^.

Within the circulation, cfDNA is rapidly degraded by nucleases, such as DNA fragmentation factor subunit beta (DFFB), intracellular deoxyribonuclease 1 like 3 (DNASE1L3), and deoxyribonuclease 1 (DNASE1). Using nuclease-deficient mouse models, the Lo group^74,75^ showed that cfDNA fragments were first generated intracellularly using DFFB, DNASE1L3 and other nucleases. Subsequently, fragmentation occurred in the extracellular space via DNASE1 and DNASE1L3. The 10 bp periodicity seen in the size profile of cfDNA originates from the cutting of DNA within an intact nucleosomal structure. DNASE1 levels are elevated in the context of trauma and may play an immunoregulatory role by clearing high cfDNA levels^76^. It must be noted that the degradation of cfDNA by nucleases only plays a partial role in cfDNA elimination^77^ and it is postulated that there are other unknown mechanisms by which cfDNA is cleared.

From circulation, cfDNA is eliminated via the liver, spleen, and kidneys. Most cfDNA (71–85%) is cleared by the liver^69^. Kupffer cells in the liver and macrophages in the spleen are responsible for trapping and clearing DNA and nucleosomes^78^. The kidneys play a smaller role. The presence of plasma-derived cfDNA in urine suggests some clearance; however, patients with chronic renal failure do not have higher levels of cfDNA^66^.

## Circulating DNA in IBD

Although there are increasing data in health and oncology, our understanding of cfDNA in inflammatory diseases, pertinently in IBD, is still at an early stage. In health, circulating cfDNA is derived mainly from the apoptosis of haematopoietic lineage cells with minimal contributions from other tissues. As discussed earlier, the short half-life suggests a tightly maintained system of degradation and filtration that limits their potential pathogenic effects^77^.

**Figure 2.**
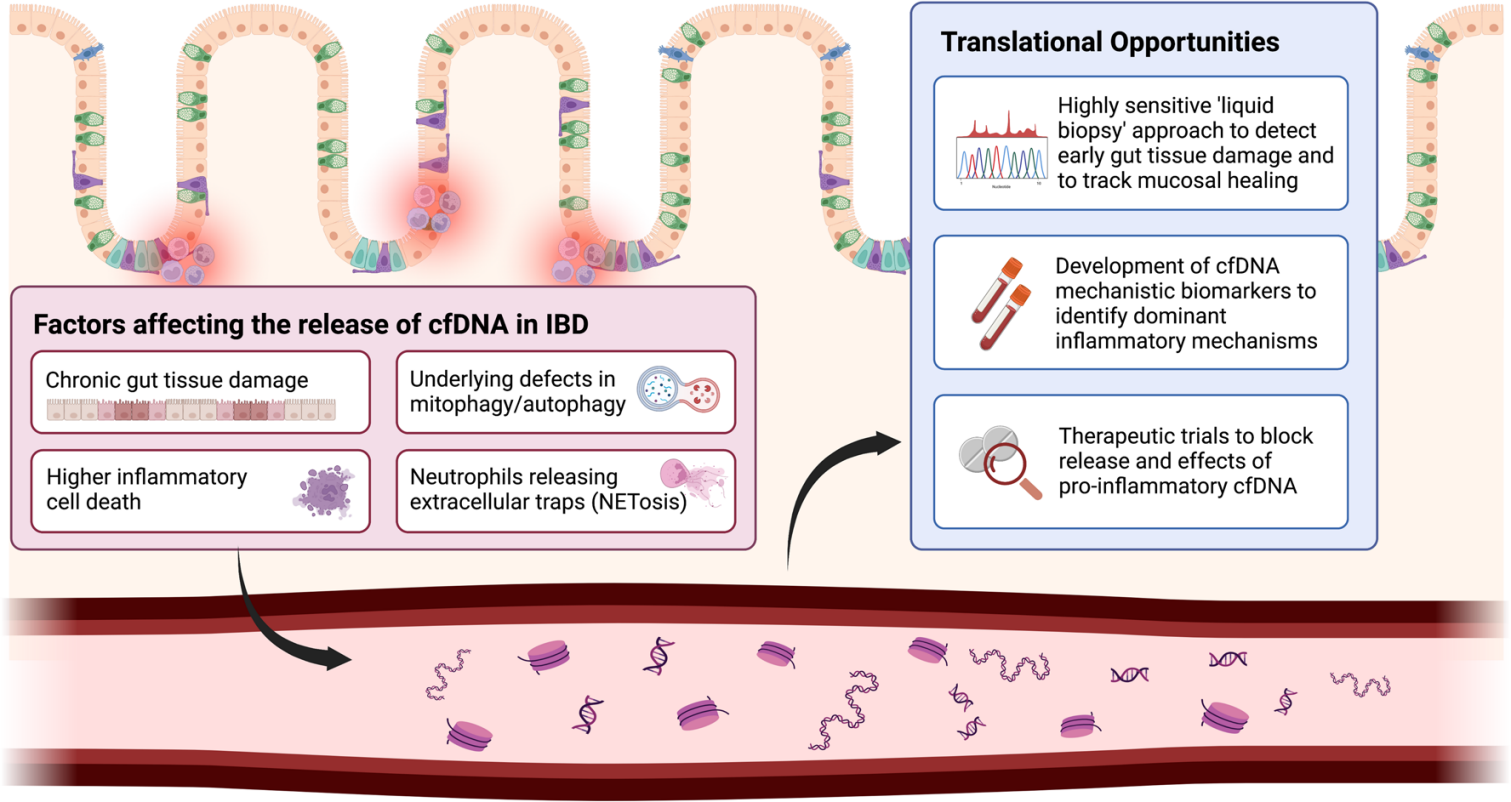
Factors affecting the release of cfDNA in IBD and translational opportunities.

Two other factors have been demonstrated to be involved in inflammatory disease, such as IBD. First, the concentration levels of circulating cfDNA were significantly higher during active systemic inflammation. Second, the underlying conditions may contribute to the dysregulation of cfDNA release (i.e. higher non-apoptotic cell death, release from the inflamed gut), pro-inflammatory qualitative changes in cfDNA (shorter fragment sizes, higher oxidised mitochondrial DNA in the setting of impaired cellular mitophagy), and impaired/overwhelmed clearance mechanisms. Traditional views of cfDNA as an epiphenomenon have been challenged by rapid advances in sequencing technologies that are more affordable and require much lower DNA concentrations, the simultaneous expansion of disease-related datasets, and the development of simple methods to stabilise cfDNA during blood sampling. Here we present key conceptual developments that will be of translational relevance in IBD (Figure 2).

### Circulating DNA as mechanistic biomarkers of IBD

In active IBD, both total circulating cfDNA and mitochondrial cfDNA levels are elevated^2,79^. In our study, patients with acute severe IBD have significantly higher levels of mitochondrial DNA; this correlation with more severe illness is in line with other diseases such as COVID-19 and trauma. However, unlike advances in oncology, where the clinical utility of cfDNA is geared toward the detection of cancer, crude plasma cfDNA concentration may not be useful for predicting the level of systemic or mucosal inflammation in IBD. Rather its use may be more relevant as a biomarker to identify dominant mechanisms (mechanistic biomarkers) in a heterogeneous complex disease, such as IBD, and potentially stratify patients to relevant therapeutic interventions.

In SLE, higher neutrophil release of the oxidised and more pro-inflammatory form of mitochondrial DNA is linked to defective mitophagy, a process that is also highly relevant in IBD. In this context, the role of mitochondrial cfDNA is more defined, given the increasing lines of evidence to implicate mitochondrial dysfunction in IBD^80^. Two Phase 2b randomised controlled trials on the use of oral mitochondrial antioxidant therapy in adults and children with UC (Marvel and MiniMarvel ClinicalTrials.gov Identifier: NCT04276740^81^) will incorporate the quantification of plasma mitochondrial cfDNA into the study design. A further study involving 1500 IBD patients will measure cfDNA and assess its correlation with mucosal healing in IBD (MUSIC study; ClinicalTrials.gov Identifier NCT04760964^82,83^).

### Finding new therapeutics from the role of cfDNA in inflammation

As discussed, nucleic acid sensors play a clear role, given that immune cells can take up cfDNA fragments with consequential effects. Earlier work has shown that this effect is not didactic, but more complex and dependent on features such as specific DNA sequence, fragment size, and structural modification, for example, oxidation of mitochondrial DNA. The earliest work was based on the study of CpG oligonucleotides (ODNs), in which three different classes of ODNs were characterised by their structure and pro-inflammatory effects. Class A ODNs are multimeric structures containing central unmethylated CpG motifs that accumulate in early endosomes, where they induce IFNα production by plasmacytoid dendritic cells (pDC) and can trigger B cell proliferation. In contrast, class B ODNs are monomeric, contain one or more unmethylated CpG motifs, and accumulate in late endosomes, where they induce pDC maturation via TLR9 signalling. Class C ODNs combine the features of class A and class B ODNs, accumulate in both early and late endosomes, and induce activation and maturation of pDCs^84^.

The triggering of PRR activation by host and bacterial DNA can have either pro- or anti-inflammatory effects depending on the tissue- and disease context. In a mouse model of colitis, bacterial CpG worsens the disease via TLR9 activation^85,86^. On the other hand, several studies have also shown the potential anti-inflammatory effect of TLR9-activation. Rachmilewitz *et al.* described synthetic Class B ODNs with multiple TCG repeats containing unmethylated CpG motifs that suppressed TNFα and IL1β generation in *ex vivo* cultured colonoscopy biopsies from UC patients^87^. This study also showed that the anti-inflammatory effect of certain unmethylated CpG ODNs depends on the exact sequence and that methylation of the CpG repeat abolished the anti-inflammatory potential. In 2017, Liu *et al.* described the immunosuppressive effects of a TLR-antagonistic CpG ODN by inhibiting innate immune signalling via TLR3 and 7/8 in a TLR9-independent manner^88^. The CpG ODN cobitolimod contains a single unmethylated CpG motif and has been shown to induce anti-inflammatory responses via TLR9^5^.

In addition to synthetic CpG-ODNs, the anti-inflammatory potential of cfDNA has also been described; however, the exact mechanisms underlying this effect have not yet been elucidated. In this context, two studies showed that prophylactic administration of cfDNA from colitic into healthy mice was protective against DSS-induced colitis^89,90^. A recent study by Constantinovits *et al.* found that injecting healthy mice with colitis-derived cfDNA before the induction of DSS-colitis changed the expression of several autophagy and inflammatory cytokines in a clinically favourable manner at the gene and protein levels^91^.

Other potential therapeutic pathways will come from the better characterisation of cfDNA and its specific effects on inflammation. This includes how blood cfDNA is released, removed from circulation and/or its downstream effects on inflammatory pathways. Notably, these studies can be run in parallel with mechanistic studies to accelerate drug development timelines. One example is our current study on the efficacy of mitochondrial antioxidant therapy in UC, where one postulated mechanism of action is by reducing the release of mitochondrial cfDNA from gut mitochondrial damage^92^. In IBD, one of the challenges in the development of therapeutic antisense oligonucleotides (ASO) that inhibit or modulate gene targets via RNA is the abundant presence of nucleases in plasma and tissue that rapidly degrade ASOs^93^.

### Development of the next generation highly specific biomarker of gut damage in IBD

A second development relates to the ability to leverage new sequencing technologies and analytical methods to identify the tissue origin of cfDNA. More recently, Esfahani *et al.* showed that the origin of cfDNA can be inferred from fragmentation profiles of cfDNA^94^. Circulating cfDNA molecules are primarily nucleosome-associated fragments; they represent distinctive chromatin configurations of the nuclear genome of cells from which they originate. Specifically, genomic regions densely associated with nucleosomal complexes are generally protected against the action of various endonucleases, whereas open chromatin is not. Hence, specific chromatin fragmentation features are potentially useful for the classification of tissue of origin by cfDNA profiling, which includes fragmentation profiles of lower-depth sequencing coverage and disruption of nucleosome positioning near transcription start sites in groups of genes relevant to a particular tissue site.

Such advances can be directly exploited to find very sensitive biomarkers of gut damage in IBD with realistic clinical applications: to track mucosal healing in IBD, to screen and detect pre-IBD in high-risk groups (i.e. those with a strong family history) and even to identify precancerous changes during IBD surveillance. From a theoretical perspective, this is likely to be superior to current clinical biomarkers such as calprotectin which relies on protein release into the gut from neutrophils or C-reactive protein produced by hepatocytes during systemic inflammation. If these technologies are proven effective, they could be compared against the current gold standard of colonoscopy, potentially decreasing the need for invasive assessment and improving upon the current non-invasive blood and stool tests used in clinics.

Using the Tabula Sapiens transcriptomic cell atlas and Human Protein Atlas RNA consensus dataset, Vorperian *et al.* generated cell-type signature scores, which allow the inference of cell types that contribute to cell-free RNA for a variety of diseases^95^. Here, they showed that loss of cell-free RNA (cfRNA)-specific signatures from tubular cells and neurons from the kidneys and brain are associated with chronic kidney disease and Alzheimer’s disease, respectively, whereas increased hepatocyte-specific cfRNA signatures are linked to hepatic steatosis^96^. Another study by the same group showed that cfRNA signatures could predict the development of preeclampsia during pregnancy^97^. This exciting progress has provided a conceptual platform for cfDNA in IBD, although much work remains to be done.

**Box 2. cfDNA in IBD–Current questions and potential translational impact**

**1. Does active IBD result in higher cfDNA release with functional relevance to the development of chronic inflammation?**

**2. Can advances in technologies allow very sensitive detection of gut inflammation and prioritisation of inflammatory mechanisms using liquid biopsy approach?**

## Conclusion

The understanding of the role of cfDNA in inflammation, in parallel with the development of technologies to accurately detect their presence, origins, and relationships with underlying inflammatory mechanisms, is likely to represent significant translational advances in the next 10 years. The use of nucleic acid-based therapies and technologies is already in the clinical mainstream – in vaccines, in ‘liquid biopsies’ in oncology, and prenatal diagnostics. In IBD, this approach is a new major step toward realising the potential of Precision Medicine. Incorporating knowledge from cellular pathophysiology, which often forms the basis of disease, into a liquid biopsy would more loosely match the resolution afforded by invasive procedures that can enable stratification and the discovery of a potentially new class of treatments in IBD.

**Box 3. Definitions**

**Nucleic acids** – A class of macromolecules found in all cells and viruses composed of nucleotides that encode information and the two major classes are DNA and RNA.

**DNA** – Deoxyribonucleic acid. Double-stranded helical structure composed of one of the four nucleotide bases (adenine, thymine, cytosine, guanine) first described by Watson and Crick, allowing for stability of genomic information and ability for repair and replication with high accuracy.

**RNA** – Ribonucleic acid. In contrast to DNA, RNA is single stranded, making it more prone to mutation and degradation. RNA has no thymine (T) but instead is replaced by uracil (U) as one of its four component nucleotides. DNA is transcribed into messenger RNA which is exported from the nucleus leading to subsequent protein expression.

**Nucleosome -**A nucleosome is the basic structural unit of DNA packaging in eukaryotes. The structure of a nucleosome consists of a segment of DNA wound around eight histone proteins and resembles thread wrapped around a spool.

**Cell-free DNA** – All non-encapsulated DNA fragments found in blood plasma.

**Liquid Biopsies** – The genomic analyses of cell-free DNA that circulates in the blood can be used in prenatal testing, oncology, and monitoring organ transplant recipients.

**Mitochondria** – Organelle found in large numbers in most cells where biochemical processes of respiration and energy production occur.

**Mitochondrial DNA** – 16.6 kilobases in length encoding for 13 proteins (ATP synthase, cytochrome c oxidase, cytochrome b, NADH dehydrogenase). 2 ribosomal RNAs (12S and 16S), 22 transfer RNAs – all of which involved in the oxidative phosphorylation process. Its genome is rich in unmethylated CpG-like bacteria. CpGs in human genomic DNA, conversely, are usually methylated as a means of controlling gene expression.

**Damage-associated molecular patterns (DAMPs)** – Host-derived endogenous signals that are released during tissue damage and cellular stress in many human inflammatory conditions.

**Pathogen-associated molecular patterns (PAMPs)** – Molecular signatures conserved by bacteria but not present in the host.

**Pattern recognition receptors (PRRs)** – Germline encoded host sensors which help detect molecular signatures that lead to an inflammatory response. These molecular signatures can be classified as either PAMPs or DAMPs.

**Nucleic acid sensors -**Toll-like receptor 9 (TLR9), cyclic GMP-AMP synthase (cGAS) and Absent in Melanoma 2 (AIM2)
